# Methylenetetrahydrofolate reductase genetic polymorphisms and toxicity to 5-FU-based chemoradiation in rectal cancer

**DOI:** 10.1038/bjc.2011.442

**Published:** 2011-11-01

**Authors:** F Thomas, A A Motsinger-Reif, J M Hoskins, A Dvorak, S Roy, A Alyasiri, R J Myerson, J W Fleshman, B R Tan, H L McLeod

**Affiliations:** 1 UNC Institute for Pharmacogenomics and Individualized Therapy, University of North Carolina, Chapel Hill, NC, USA; 2 EA3035, Université de Toulouse, Institut Claudius Regaud, 20-24, rue du Pont St Pierre, Toulouse cedex 31052, France; 3 Department of Statistics, North Carolina State University, Raleigh, NC, USA; 4 Bioinformatics Research Center, North Carolina State University, Raleigh, NC, USA; 5 Department of Medicine, Washington University School of Medicine, St Louis, MO, USA; 6 Department of Radiation Oncology, Washington University School of Medicine, St Louis, MO, USA; 7 Department of Surgery, Washington University School of Medicine, St Louis, MO, USA

**Keywords:** MTHFR polymorphisms, 5-FU, mucositis, diarrhoea, rectal cancer, neoadjuvant chemoradiation

## Abstract

**Background:**

There is a large degree of variation in tumour response and host toxicities associated with neoadjuvant chemoradiation for rectal cancer patients. We performed a complimentary pharmacogenetic study to investigate germline polymorphisms of genes involved in 5-fluorouracil (5-FU) and irinotecan pathways and their potential association with clinical outcomes and toxicities from neoadjuvant chemoradiation in patients with rectal cancer treated in a prospective genotype-directed study.

**Methods:**

The germline DNA of 131 patients was genotyped for 10 variants in *TYMS*, *MTHFR*, *DPYD*, *UGT1A1*, *ABCC1* and *SLCO1B1* genes. Ninety-six patients were treated with 5-FU/radiotherapy (RT) and 35 received 5-FU/RT/irinotecan. Relationships between genetic variants and adverse events, tumour response, overall and disease-free survivals were assessed.

**Results:**

*MTHFR* 1298A>C and *MTHFR* diplotypes (for 677C>T and 1298A>C) were associated with chemoradiation-related toxicity when 5-FU was used alone. *MTHFR* haplotypes (677C–1298C) and diplotypes (CA–TA and TA–TA) showed, respectively, a protective and a negative effect on the incidence of severe diarrhoea or mucositis. No association was observed between genetic markers and drug response.

**Conclusion:**

*MTHFR* polymorphisms can potentially predict toxicity in patients treated with 5-FU as a single chemotherapeutic drug.

Neoadjuvant fluoropyrimidine-based chemoradiation is currently the standard therapy for patients with locally advanced rectal cancer (Sauer *et al*, 2004; Roh *et al*, 2009). Preoperative treatment was associated with lower risks of local recurrence and lower toxicities compared with radiotherapy (RT) alone (Bosset *et al*, 2006; Gerard *et al*, 2006; Braendengen *et al*, 2008) or postoperative chemoradiation CRT (Sauer *et al*, 2004; Roh *et al*, 2009). Pathologic downstaging (DS) or a pathologic complete response (pCR) after preoperative CRT has been correlated with improved survival, decreased recurrence and a higher rate of sphincter-preserving surgeries (Theodoropoulos *et al*, 2002; Valentini *et al*, 2002; Garcia-Aguilar *et al*, 2003; Crane *et al*, 2003b). The pCR and the DS rates observed with 5-fluorouracil (5-FU)-based CRT are 10–20% (Sauer *et al*, 2004; Bosset *et al*, 2005) and ∼50–65% (Garcia-Aguilar *et al*, 2003; Crane *et al*, 2003a), respectively.

The efficacy of other drugs available in colon cancer treatment, including capecitabine (Dunst *et al*, 2002), oxaliplatin (Aschele *et al*, 2009; Gerard *et al*, 2010), irinotecan (Mohiuddin *et al*, 2006), cetuximab (Bertolini *et al*, 2009) or bevacizumab (Willett *et al*, 2009), has been evaluated in this setting with various results in terms of tumour response but to date, no benefit on survival was observed compared with the current 5-FU regimen. In the context of multiple treatment possibilities, the identification of predictive markers of response to chemoradiotherapy treatment is a challenging approach for drug selection in order to obtain the best clinical benefit while minimising the side effects for each patient.

5-Fluorouracil is an antimetabolite of the pyrimidine analogue type that inhibits DNA and RNA synthesis (Figure 1). Its main mechanism of action consists of inhibition of thymidylate synthase (TS) through an active metabolite, fluorodeoxyuridine monophosphate (FdUMP), which forms an inactive ternary complex with TS and 5–10-methylenetetrahydrofolate (MTHF). Optimal inhibition of TS requires an elevated level of MTHF, which is regulated by the enzyme methylenetetrahydrofolate reductase (MTHFR). As a consequence, both TS and MTHFR activities are presumed to be major determinants of 5-FU clinical response. Genetic polymorphisms with functional impact on the activity and/or expression of both enzymes have been described.

A polymorphism within the promoter enhancer region (TSER) of *TYMS* (the TS gene), consisting of tandem repeats of 28 bp, has been implicated in modulating TS mRNA expression (Kaneda *et al*, 1987; Horie *et al*, 1995) and TS mRNA translational efficiency (Kawakami *et al*, 2001). The most common alleles are the double repeat (2R, TSER^*^2) and triple repeat (3R, TSER^*^3). *In vitro* and *in vivo* studies have shown that increasing the number of repeats (from 2R to 3R) leads to a stepwise increase in TS expression (Horie *et al*, 1995; Kawakami *et al*, 1999; Pullarkat *et al*, 2001).

TSER^*^3 homozygosity appears to be associated with a lower response to neoadjuvant 5-FU-based CRT for patients with rectal cancer (Villafranca *et al*, 2001; Spindler *et al*, 2007). Thus, we conducted a prospective non-randomised single-institution phase II study using *TYMS* genotyping to direct neoadjuvant CRT for patients with locally advanced and metastatic rectal cancer. Patients with germline TSER^*^2/^*^2 or TSER^*^2/^*^3, deemed ‘good risk’ for a favourable response to 5-FU, were treated with standard CRT. Poor-risk patients with a TSER^*^3/^*^3 or TSER^*^3/^*^4 genotypes who were unlikely to derive significant benefit from 5-FU chemotherapy, were treated with irinotecan in addition to standard 5-FU/CRT. The clinical results of this study have been published (Tan *et al*, 2011) and showed that patients of the poor-risk group can achieve similar tumour DS and pCR rates to patients of the good-risk group when irinotecan was added to the 5-FU/RT regimen. However, in each genotype/treatment group, there was inter-individual variability in tumour response and treatment toxicities. Several studies regarding pharmacogenetic predictors of 5-FU pharmacodynamics have been conducted in colorectal cancer patients with inconsistent results (Etienne *et al*, 2004; Ruzzo *et al*, 2007, 2008; Capitain *et al*, 2008; Gusella *et al*, 2009). We performed a complimentary retrospective pharmacogenetic study investigating germline polymorphisms of genes involved in 5-FU and irinotecan pathways to assess their putative role in the prediction of outcome or toxicity in rectal cancer patients receiving 5-FU-based chemoradiation.

## Materials and methods

### Patients and treatment

All patients were included in a clinical phase II study using *TYMS* genotyping to direct neoadjuvant CRT for patients with rectal cancer (Tan *et al*, 2011). Patients were eligible to participate in the study if they were 18 years old or older, with biopsy-proven clinical T3/T4, N0-2, M0-1 adenocarcinoma of the rectum and a Karnofsky performance status of ⩾60%. Patients with metastatic disease, whose primary tumours were deemed resectable, were also eligible. Patients who qualified had adequate haematologic (absolute neutrophil count 1500 mm^–3^, platelets count ⩾100 000 mm^–3^), renal (creatinine ⩽2.0 mg dl^–1^) and hepatic functions with total bilirubin ⩽2.0 mg dl^–1^, SGOT and alkaline phosphatase ⩽2 × upper limit of normal. Exclusion criteria included prior pelvic radiation, prior malignancies in the past 5 years except for skin cancer and *in situ* cervical cancers and known allergy to 5-FU or irinotecan. This study was approved by the Institutional Review Board at Washington University School of Medicine and informed consent was obtained from all participants before enrolment.

Before treatment, clinical staging was performed, blood samples were obtained and TSER polymorphisms were evaluated using a previously described PCR-based assay (Marsh *et al*, 1999). Patients carrying at least one ^*^2 allele (TSER^*^2/^*^2, ^*^2/^*^3, or ^*^2/^*^4) were assigned to the good-risk genotype group (i.e. group 1) and treated with standard preoperative CRT. Radiotherapy consisted of a total of 45–50.4 Gy delivered in 25–28 fractions (180–200 cGy per fraction) by a multiple field technique using image-guided radiotherapy with radiotherapy target volume consistent with the Radiation Therapy Oncology Group consensus guidelines (Myerson *et al*, 2009). Concurrent continuous intravenous infusion of 5-FU at a dose of 225 mg m^–2^ per day was administered throughout radiation without leucovorin. Patients with TSER^*^3/^*^3 or TSER^*^3/^*^4 genotypes were assigned to the poor-risk genotype group (i.e. group 2) and treated with weekly intravenous irinotecan at 50 mg m^–2^ for 5 weeks in addition to standard CRT identical to the treatment in the good-risk group. Clinical restaging and resection of the primary rectal lesion were performed 6–10 weeks after completion of preoperative CRT.

### Evaluation of patients

Baseline clinical tumour staging, using rigid proctoscopy, transrectal ultrasound (TRUS), spiral computed tomography (CT) or magnetic resonance imaging (MRI), were performed within 28 days of enrolment. Clinical restaging with TRUS, CT or MRI was repeated before resection. The surgical procedure performed was at the discretion of the treating surgical oncologist. Standardised institutional pathology examinations were done, and the pathologic staging as well as the extent of residual tumour in the resected specimen were classified using the American Joint Committee on Cancer version 6. Tumour DS was defined as a decrease in the T stage of the primary tumour by at least 1. Tumour complete response (ypT0) was defined as the absence of any viable tumour in the rectum. Toxicities were graded according to the National Cancer Institute Common Toxicity Criteria (NCI-CTC) version 2.0. Recurrence and survival were also monitored.

### Genotype analysis

Genomic DNA was isolated from whole blood using the Puregene DNA isolation kit (Qiagen, Hilden, Germany).

Based on previous published studies, the 5-FU-related genetic polymorphisms selected for testing were *TYMS* TSER^*^3 G>C (rs2853542), two intronic SNPs c.205+117G>C (rs2853533) and c.280-499G>A (rs2847153), *MTHFR* 677C>T (rs1801133, currently referred as c.665C>T) and 1298A>C (rs1801131, currently referred as c.1286A>C) and DPYD^*^2A (rs3918290).

For irinotecan, in addition to the *UGT1A1* (TA)_*n*_ (rs8175347), we genotyped samples for the *UGT1A1* –3156G>A SNP (rs10929302, currently referred as c.862-9898G>A), and two polymorphisms in transporters, *ABCC1* c.1474-48C>T (rs3765129) and *SLCO1B1 c.*388A>G (rs2306283) that have been described to have an effect on irinotecan toxicity (Innocenti *et al*, 2009).

For *TYMS* TSER^*^3 G>C SNP, the PCR and the RFLP were performed as described previously (Thomas *et al*, 2010). The *TYMS* TSER^*^3 G>C SNP leads to a change of a critical residue in the upstream stimulatory factor E-box consensus element (CACTT**G**>CACTT**C**). The number of theoretical E-box-binding sites likely to bind USF proteins was deduced based on the *TYMS* G>C genotype: one E-box for genotypes 2RC/3RC or 2RC/2RG, two E-boxes for genotypes 2RG/2RG or 2RG/3RC or 3RC/3RC or 2RC/3RG, three E-boxes for 2RG/3RG or 3RC/3RG, four E-boxes for 3RG/3RG or 2RG/4R or 3RC/4R and five E-boxes for 3RG/4R. *TYMS* genotypes with ⩽2 E-boxes were classified as ‘low expression of TS’ and ⩾3 were classified as ‘high expression of TS’ as proposed by Kawakami and Watanabe (2003).

Genotypes for *TYMS* c.205+117G>C, *TYMS* c.280-499G>A and SLCO1B1^*^1b 388A>G were determined using allelic discrimination with TaqMan SNP Genotyping assays (Applied Biosystems, Foster City, CA, USA) C_26612339_10 and C_1637481_10 and C_1901697_20, respectively.

Genotyping for *MTHFR* 667C>T, *MTHFR* 1298A>C, DPYD^*^2A, *UGT1A1* (TA)_*n*_ and *UGT1A1* –3156G>A were determined using pyrosequencing as previously described (Marsh *et al*, 2005; McLeod *et al*, 2010). *ABCC1* IVS11 –48C>T was genotyped using pyrosequencing (Marsh *et al*, 2005) with the following primers: biotinylated-forward 5′-AGCATGGTGAAACCCATCT-3′ reverse 5′-TCAGCTTGATCCGATTGTCTT-3′ and sequencing 5′-GGGCGACCCTGGGAT-3′.

### Statistics

Each SNP was tested for deviation from Hardy–Weinberg equilibrium. Since not all SNPs are biallelic, binomial expansions of the equation were used to compute expected values. *χ*^2^ tests of association were used if the expected value of each cell was >5, and Fisher's exact tests were used when the cells were too sparse. Additionally, tests for deviation from Hardy–Weinberg proportions were performed two ways: first on the entire cohort (*n*=131) and second on Caucasian samples only (*n*=111, other racial groups did not have enough individuals for stratified analysis).

Relationships between genetic variants and the incidence of grade 3–4 toxicity, tumour response (measured by DS and ypT0 rates), overall and disease-free survivals were assessed.

The association analyses were performed for each genotype variable, using categorical genotypic encodings. For each genotype variable and outcome combination, traditional tests of hypotheses were performed based on the data types in the model, and whether the data met parametric assumptions. When possible, parametric tests were performed to increase power, and non-parametric tests were used when statistical assumptions were not met. Contingency table analyses and proportional hazards analyses were used for the categorical outcomes and time-to-event outcomes, respectively. When significant associations were found, corresponding odds ratios (ORs) and their 95% confidence intervals (95% CIs) were calculated. Permutation tests were performed to correct for multiple testing for each outcome leading to different cutoff *P*-values for each outcome. Additionally, multiple logistic regression analysis was performed for each of the four outcomes against the different treatments using both markers and treatment as factor. The model was logit(*y*)=intercept+marker+treatment+marker × treatment. If no significant results were seen in the multiple regression analyses, details are not shown. Analysis was performed in Stata v.11 (StataCorp LP, College Station, TX, USA; http://www.stata.org) and R (http://www.r-project.org and http://cran.r-project.org/doc/FAQ/R-FAQ.html). Haplotypes were determined with Shape-IT, publicly available at http://www.griv.org/shapeit/ (Delaneau *et al*, 2008).

## Results

### Patients and treatment outcome

There were 131 patients treated and evaluable for assessment of toxicity, overall survival (OS) and recurrence-free survival (RFS) (both considered as intent to treat). Among the 131 patients, 96 were assigned to group 1 based on their *TYMS* TSER genotype and were treated with 5-FU+RT and 35 were in the group 2 and were treated with 5-FU+RT+irinotecan. As shown in Table 1, a total of 10 patients were inevaluable for tumour DS and ypT0 evaluation, mostly for surgery issues. The clinical results have been previously published (Tan *et al*, 2011) and are summarised in Table 1. Briefly, in group 1, the DS and ypT0 rates were 64.4% and 20%, respectively. Grade 3 or 4 toxicities occurred in 30.2% of patients.

In group 2, which was treated with 5-FU+RT+irinotecan, 19 of the 35 (54.3%) patients experienced grade 3–4 toxicities and the DS and ypT0 rates were 64.5% and 41.9%, respectively. To assess the impact of the genotype on treatment-specific toxicity, the existence of grade 3/4 diarrhoea and/or mucositis has been considered together for patients treated with 5-FU+RT (Table 1).

### Genotyping

The genotyping results are presented in Table 2. For the *TYMS* TSER^*^3 G>C SNP, five alleles were identified: 2RC, 2RG, 3RC, 3RG and 4R. Among the patients carrying the 3RC allele, two displayed an unexpected size of the 3R allele that was due to a 6-bp insertion (CCCCCG) in the second repeat of the 3R allele. This particular finding has been recently reported (Thomas *et al*, 2010). For the current pharmacogenetic study, these two patients have been considered as carrying the 3RC allele because their small number did not allow studying them separately. Genotypes were first grouped based on the number of E-boxes (as shown in Table 2) and then into ‘low expression’ *vs* ‘high expression’ of TS for statistical analyses. It is noteworthy that the repartition of these genotypes is biased in each group since the groups have been created based on the *TYMS* TSER polymorphism (^*^2/^*^2+^*^2/^*^3+^*^2/^*^4 *vs* ^*^3/^*^3+^*^3/^*^4). No DPYD*2A variant was identified in the studied cohort of patients. The observed genotypes were in agreement with the Hardy–Weinberg equilibrium in the Caucasian population. The resulting *P*-values from these tests are shown in Table 2 for the 111 Caucasians out of the 131 patients.

Haplotypes for *MTHFR* 677C>T and 1298A>C were also analysed. Four haplotype identities were observed; although the TC haplotype had a very low frequency (0.38%) compared with CA (35.9%), CC (32.4%) and TA (31.3%) haplotypes. Linkage disequilibrium was weak between these two SNPs with *r*^2^=0.27 in Caucasians, which is similar to that reported in the HapMap Caucasian population (*r*^2^=0.22), and *r*^2^=0.20 in all patients (http://hapmap.ncbi.nlm.nih.gov/).

### Genetic polymorphisms and drug response to 5-FU

Drug response was assessed with four variables: tumour DS, ypT0, OS and RFS. The following genetic markers were tested for associations with drug response: *TYMS* 3R G>C SNP, the number of *TYMS* E-boxes, TS expression, the two intronic *TYMS* SNPs (rs2847153 and rs2853533), *MTHFR* 677C>T, and 1298A>C, *MTHFR* diplotype for 677C>T and 1298A>C and *MTHFR* haplotypes. The analyses were first performed in the group 1 to assess the impact of genetic markers on response to 5-FU alone. All markers except those regarding the TSER region of *TYMS* (*TYMS* 3R G>C SNP, the number of *TYMS* E-boxes and the TS expression) were also tested in the whole group (*n*=131) representing a mix of patients treated with 5-FU alone or in combination with irinotecan. None of the genotypes was significantly associated with drug response in group 1 or the whole group.

### Genetic polymorphisms and 5-FU toxicity

The same genetic markers were tested for associations with 5-FU toxicity in group 1. By considering the general grade 3–4 toxicity, one *MTHFR* diplotype (CA–TA) was significantly related to a higher rate of grade 3–4 toxic events (*P*=0.006). The *MTHFR* genotypes, haplotypes and diplotypes were then analysed with regards to 5-FU-specific toxicity such as diarrhoea and mucositis. The results are presented in Table 3 and Figure 2. The *MTHFR* 1298A>C genotype was significantly associated with grade 3–4 diarrhoea and/or mucositis (*P*=0.005), with patients with the A/A genotype having a higher risk of toxicity (OR=4.71; 95% CI=1.63, 13.59) compared to patients with the A/C or C/C genotype (Figure 2A). Although not significant, the 677C/C genotype tended to be protective from G3–4 diarrhoea and/or mucositis, as shown in Figure 2B. Consistent with these results, the *MTHFR* CC (677C–1298C) haplotype was associated with a lower incidence of G3–4 diarrhoea and/or mucositis (Figure 2D; *P*=0.005; OR=0.21; 95% CI=0.074, 0.61). The *MTHFR* diplotype showed that the patients carrying the CA–TA and TA–TA diplotypes had a higher risk (OR=7.75; 95% CI=2.67, 22.47) of developing grade 3–4 diarrhoea and/or mucositis (*P*=0.003; see Figure 2C). These relationships between *MTHFR* markers and grade 3–4 diarrhoea and/or mucositis were not observed in group 2, which was treated with 5-FU+RT+irinotecan.

### Genetic polymorphisms and drug response to 5-FU+irinotecan

Associations between *UGT1A1* (TA)_*n*_, *UGT1A1* –3156G>A, SLCO1B1*1b 388A>G and *ABCC1* IVS11 –48C>T genotypes and drug response (tumour DS, ypT0, OS and RFS) to 5-FU+irinotecan were evaluated for the 35 patients of group 2. None of these markers was significantly associated with drug response.

### Genetic polymorphisms and 5-FU+irinotecan toxicity

The impact of *UGT1A1* (TA)_*n*_, *UGT1A1* –3156G>A, SLCO1B1^*^1b 388A>G and *ABCC1* IVS11 –48C>T genotypes was evaluated on toxicity related to 5-FU+irinotecan, for example, in the 35 patients of group 2. None of these markers was associated with grade 3–4 overall toxicity.

## Discussion

The main finding of this study is that *MTHFR* polymorphisms are associated with 5-FU-specific toxicity when 5-FU is used alone. Patients carrying the 1298A/A genotype have a higher risk of developing grade 3–4 diarrhoea or mucositis compared to patients with the A/C or C/C genotype when treated with 5-FU alone but this risk was not observed in patients treated with 5-FU and irinotecan. By considering the *MTHFR* haplotypes, we observed that only 10% of the patients carrying the 677C–1298C haplotype experienced grade 3–4 diarrhoea or mucositis compared with 36% of patients with other haplotypes, suggesting that the CC haplotype confers a protective effect. Consistent with these observations, two *MTHFR* diplotypes CA–TA and TA–TA (homozygous wild type for 1298A>C and at least one variant allele for 677C>T) were predictive of a higher rate of grade 3–4 diarrhoea or mucositis. Opposite findings have been published by Capitain *et al* (2008), regarding the role of *MTHFR* 1298A>C polymorphism on 5-FU toxicity in colorectal patients. They found that homozygosity for *MTHFR* 1298C/C confers a higher risk of grade 3–4 toxic events. However, the effect of this polymorphism was not assessed on toxicity such as diarrhoea or mucositis and the percentage of patients undergoing serious toxic events was lower than in the present study. Moreover, the regimen used in their study included leucovorin whereas the patients in our study did not receive leucovorin in combination with 5-FU. This raises the question of the results inconsistency regarding the role of *MTHFR* polymorphisms as predictive markers of treatment outcome and toxicity that has been published in colorectal patients (De Mattia and Toffoli, 2009). It is hypothesised that *MTHFR* polymorphisms may augment the cytotoxic activity of 5-FU by increasing intracellular concentrations of 5,10-methylentetrahydrofolate and then enhancing the formation and stability of the ternary inhibitory complex (Bagley and Selhub, 1998; Weisberg *et al*, 1998). *MTHFR* 1298C and 677T variants are then theoretically associated with a higher cytotoxicity. The trend we observed for 677T being associated with a higher incidence of grade 3–4 diarrhoea or mucositis is in agreement with this hypothesis; on the contrary, the association observed between *MTHFR* 1298A variant and toxicity is opposite to what was expected. However, most of the studies investigating the role of *MTHFR* polymorphisms in colorectal patients treated with fluoropyrimidine-based chemotherapy have yielded conflicting results (reviewed in De Mattia and Toffoli, 2009). Among the reasons for these inconsistencies, we can mention the variety of drugs (oxaliplatin, irinotecan, etc.) co-administered with the fluoropyrimidine, different clinical settings (adjuvant, neoadjuvant, first- and second-line palliative chemotherapy), the route of administration for 5-FU (bolus and infusion) and finally the addition of leucovorin. All these factors might influence the associations between *MTHFR* polymorphisms and fluoropyrimidine activity.

**Figure 1:**
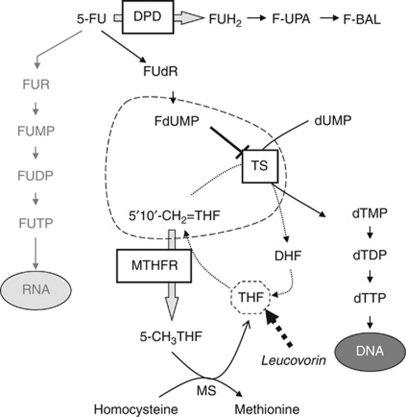
5-Fluorouracil pathway. Abbreviations: DPD=dihydropyrimidine dehydrogenase; FUH_2_=dihydrofluorouracil; F-UPA=fluoro-*β*-ureidopropionate; F-BAL=5-fluoro-*β*-alanine; FUR=fluorouridine; FUMP=fluorouridine monophosphate; FUDP=fluorouridine diphosphate; FUTP=fluorouridine triphosphate; FUdR=5-fluorodeoxyuridine; dUMP=deoxyuridine 5′-monophosphate; dTMP=deoxythymidine 5′-monophosphate; dTDP=deoxythymidine 5′-diphosphate; dTTP=deoxythymidine 5′-triphosphate; 5–10-CH_2_THF=5–10-methylenetetrahydrofolate; 5-CH_3_THF=5-methyltetrahydrofolate; THF=tetrahydrofolate; DHF=dihydrofolate; MTHFR=methylenetetrahydrolate reductase; MS=methionine synthetase.

**Figure 2:**
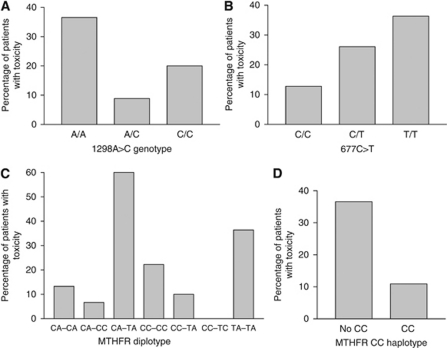
Relationships between incidence of grade 3–4 diarrhoea and/or mucositis experienced by patients treated with 5-FU/RT (group 1) with *MTHFR* 1298A>C genotype (**A**), *MTHFR* 677C>T genotype (**B**), *MTHFR* diplotype (**C**) and *MTHFR* CC haplotype (**D**).

Our study represents an ideal situation for investigating the role of *MTHFR* polymorphisms in 5-FU outcome because (i) 5-FU was given as monotherapy in group 1; (ii) no leucovorin was added to the 5-FU regimen; therefore, the pool of methylenetetrahydrofolate was directly controlled by MTHFR (see Figure 1); (iii) 5-FU cytoxicity, when given as a continuous infusion, has been shown to rely more on inhibition of TS activity and DNA synthesis than RNA inhibition (Tsujinaka *et al*, 1992; Noordhuis *et al*, 2004); and (iv) patients were chemonaive, which excludes the possibility of acquired resistance to chemotherapy. For patients treated with chemoradiation using irinotecan and 5-FU, the association of *MTHFR* genotype and toxicity could not be clearly defined. This can be due to the very small sample size (*n*=35) but we can hypothesise that the effect of MTHFR polymorphisms on 5-FU toxicity is abrogated when irinotecan is added to the regimen. Indeed, the leading cause of diarrhoea observed in group 2 is certainly irinotecan and, therefore, seems independent on MTHFR. More so, the incidence of grade 3–4 diarrhoea was high (45.7%) among patients treated with irinotecan-based chemoradiation, compared with 17.7% among those treated with 5-FU alone in our prospective study (Tan *et al*, 2011). This difference in toxicity between the two treatment groups may abrogate the effect MTHFR on toxicity among irinotecan-treated patients. Moreover, due to the trial design, groups 1 and 2 differ also from each other in terms of TYMS genotype. Therefore, we cannot exclude that the difference observed between the two groups for MTHFR effect may be due to TYMS genotype and not to different therapies.

This is the first study investigating the role of pharmacogenetics in 5-FU toxicity in rectal cancer while two studies have already looked at the influence of these genetic markers on drug response (Terrazzino *et al*, 2006; Balboa *et al*, 2010). Terrazzino *et al* (2006) showed that patients with the *MTHFR* 667T–1298A haplotype had a lower tumour regression rate compared with other haplotypes. However, among the 125 patients investigated, 25% received 5-FU as a bolus and only 36% were treated with 5-FU alone. More recently, Balboa *et al* (2010) found no relationship between *MTHFR* polymorphisms and tumour response, which is in agreement with our findings. The impact of *MTHFR* polymorphisms has also been evaluated on capecitabine toxicity in breast cancer patients (Largillier *et al*, 2006) and colorectal patients (Sharma *et al*, 2008). Although the first study did not identify any associations between *MTHFR* polymorphisms and toxicity, Sharma *et al* (2008) showed that the *MTHFR* 677T/T and 1298A/A genotypes were associated with a lower incidence of grade 2–3 overall toxicity, which is not in agreement with our findings. They also identified *MTHFR* diplotypes (CA–CC and CC–TA) that conferred a higher risk of toxicity. In our analysis, these diplotypes were not predictive of a higher incidence of toxicity. Among potential explanations for these discrepancies, we can mention that capecitabine, despite being an oral prodrug of 5-FU, presents some differences with 5-FU in the safety profile (e.g. lower incidence of stomatitis and diarrhoea, higher incidence of hand foot syndrome) (Cassidy *et al*, 2002). Also, in our study, we tested genotype associations with grade 3–4 toxicity whereas Sharma *et al* (2008) considered grade 2–3 toxic events because of low incidence of toxicity from capecitabine.

*TYMS* polymorphisms were not identified as predictive markers of drug response and toxicity in our study but it may be due to its design. Indeed, group 1 (treated with 5-FU+RT) was only composed of patients with TSER^*^2/^*^2 or TSER^*^2/^*^3 genotypes, which decreased the incidence of the TSER^*^3 G>C SNP in this group and then probably the range of TS activity. On the other hand, patients carrying the TSER^*^3/^*^3 genotype and thereby being more susceptible to carry the TSER^*^3 G>C SNP were included in group 2 and treated with 5-FU+RT+irinotecan. The addition of irinotecan increased the incidence of grade 3–4 adverse events and in particular diarrhoea. Thus, the addition of irinotecan to the regimen is a confounding factor and complicates the identification of relationships between the *TYMS* TSER^*^3 G>C SNP and 5-FU response or toxicity. For this reason, it is not possible to draw any conclusion regarding the lack of significant relationships between TYMS polymorphisms and 5-FU outcome from our study.

Regarding the pharmacogenetics of irinotecan, none of the genetic markers investigated was significantly associated with irinotecan toxicity and response. The low dose of irinotecan used in this regimen (i.e. 50 mg m^–2^) and the small number of patients treated (*n*=35) might explain the lack of associations observed (Hoskins *et al*, 2007). Indeed, homozygosity for UGT1A1*28 has been clearly identified as a risk factor for developing severe irinotecan-induced neutropenia, while heterozygous patients seem at intermediate risk (Kim and Innocenti, 2007). Innocenti *et al* (2009) showed that irinotecan-induced neutropenia can be explained in part by the UGT1A1*93, *ABCC1* IVS11 –48C>T and SLCO1B1*1b 388A>C genetic polymorphisms, but in their study, irinotecan was administered at considerably higher doses (300–350 mg m^–2^); therefore, it is not surprising that we could not reproduce these associations.

In summary, our results suggest that *MTHFR* genetic polymorphisms (particularly *MTHFR* 1298A>C and diplotype) are predictive for grade 3–4 diarrhoea or mucositis when 5-FU is administered as a single agent but not in combination with irinotecan. These findings need to be validated in a larger cohort and the results obtained in the group treated with 5-FU+irinotecan should be interpreted with caution for small sample size and confounding factors. Our study demonstrates that pharmacogenetic–pharmacodynamics studies require certain homogeneity in the selection of patients and therapy and that the presence of concomitant chemotherapeutic agents (such as irinotecan in this study) may confound the results by participating in the global pharmacodynamic events.

## Figures and Tables

**Table 1:** Treatment outcomes used as variables in the pharmacogenetic study

	**Group 1 (5-FU+radiotherapy-treated group)**	**Group 2 (5-FU+radiotherapy+irinotecan-treated group)**
Total number of patients	96	35
		
*Number of evaluable patients for*		
DS and ypT0	90	31
Overall and relapse-free survivals	96	35
Toxicity	96	35
		
Tumour DS	58 (64.4%)	20 (64.5%)
ypT0	18 (20%)	13 (41.9%)
3-year overall survival	78.2%	83.6%
3-year relapse-free survival	70.7%	68.4%
Grade 3–4 toxicity	29 (30.2%)	19 (54.3%)
Grade 3–4 diarrhoea and/or mucositis	21 (21.9%)	16 (45.7%)

Abbreviations: DS=downstaging; ypT0=tumour complete response; 5-FU=5-fluorouracil.

**Table 2:** Genotype and allele frequencies for the genetic polymorphisms tested

	**Patients**		**Allele frequency in all patients (*n*=131)**		
**Genotype**	**Group 1, *n*=96**	**Group 2, *n*=35**	**p**	**q**	**HWE *P*-value**^a^ **in Caucasians**
*TYMS* 3R G>C (number of E-boxes)					
2RC/2RG or 2RC/3RC (1)	3	0	2RG: 0.450		>0.99
2RC/3RG or 2RG/2RG or 2RG/3RC or 3RC/3RC (2)	60	12	2RC: 0.015		
2RG/3RG or 3RG/3RC (3)	32	17	3RG: 0.225		
2RG/4R or 3RC/4R or 3RG/3RG (4)	1	5	3RC: 0.298		
3RG/4R (5)	0	1	4R: 0.012		
					
*TYMS* c.280-499G>A					
GG	64	19	0.79	0.21	>0.99
GA	28	13			
AA	4	3			
					
*TYMS* c.205+117G>C					
GG	68	20			0.68
GC	25	10	0.81	0.19	
CC	2	5			
ND	1	0			
					
*MTHFR* 677C>T					
CC	39	21	0.68	0.32	0.53
CT	46	13			
TT	11	1			
					
*MTHFR* 1298A>C					
AA	41	17	0.67	0.33	0.84
AC	45	15			
CC	10	3			
					
*UGT1A1(TA)*_n_*TAA* (a28)					
(TA)_5_/(TA)_6_	0	1			0.50
(TA)_5_/(TA)_7_	3	0	(TA)_5_: 0.015		
(TA)_6_/(TA)_6_	42	16	(TA)_6_: 0.664		
(TA)_6_/(TA)_7_	41	14	(TA)_7_: 0.298		
(TA)_7_/(TA)_7_	8	3	(TA)_8_: 0.012		
(TA)_6_/(TA)_8_	1	1			
(TA)_7_/(TA)_8_	1	0			
					
*UGT1A1* –3156G>A (a93)					
GG	47	20	0.73	0.27	0.64
GA	44	12			
AA	5	3			
					
*SLCO1B1* 388A>G					
AA	29	11	0.53	0.47	>0.99
AG	46	12			
GG	21	12			
					
*ABCC1* c.1474-48C>T					
CC	74	31	0.90	0.10	>0.99
CT	19	4			
TT	1	0			
ND	2				
					
*DPYD* c.1905+1G>A					
GG	96	35	1.00	0.00	>0.99
GA	0	0			
AA	0	0			

Abbreviations: HWE=Hardy–Weinberg equilibrium; MTHFR=methylenetetrahydrofolate reductase.

a Calculated with Fisher's exact test, except for *TYMS* 3R G>C and *UGT1A1(TA)*_*n*_*TAA* that were calculated with *χ*^2^ test. The *P*-values reported are uncorrected for multiple comparisons.

**Table 3:** Associations between *MTHFR* genotypes, haplotypes and diplotypes and grade 3–4 diarrhoea and/or mucositis

	**Group 1**			**Group 2**		
	**Number of patients (%) without G3–4 diarrhoea and/or mucositis, *N*=75 (78%)**	**Number of patients (%) with G3–4 diarrhoea and/or mucositis, *N*=21 (22%)**	***P*-value** a	**Number of patients (%) without G3–4 diarrhoea and/or mucositis, *N*=19 (54%)**	**Number of patients (%) with G3–4 diarrhoea and/or mucositis, *N*=16 (46%)**	***P*-value** a
*MTHFR* 677C>T						
CC	34 (87)	5 (13)		12 (57)	9 (43)	
CT	34 (74)	12 (26)	0.138	6 (46)	7 (54)	0.851
TT	7 (64)	4 (36)		1 (100)	0 (0)	
						
*MTHFR* 1298A>C						
AA	26 (63)	15 (37)		9 (53)	8 (47)	
AC	41 (91)	4 (9)	**0.005**	9 (60)	6 (40)	0.699
CC	8 (80)	2 (20)		1 (33)	2 (67)	
						
*MTHFR* haplotype						
677C–1298A	33 (73)	12 (27)	0.329	15 (58)	11 (42)	0.700
677C–1298C	49 (89)	6 (11)	**0.005**	10 (56)	8 (44)	>0.99
677T–1298A	40 (71)	16 (29)	0.080	7 (50)	7 (50)	0.739
						
*MTHFR* diplotype						
CA–CA	13 (87)	2 (13)	**0.003**	4 (50)	4 (50)	0.766
CA–CC	14 (93)	1 (7)		7 (70)	3 (30)	
CA–TA	6 (40)	9 (60)		4 (50)	4 (50)	
CC–CC	7 (78)	2 (22)		1 (33)	2 (66)	
CC–TA	27 (90)	3 (10)		2 (40)	3 (60)	
CC–TC	1 (100)	0 (0)		0	0	
TA–TA	7 (64)	4 (36)		1 (100)	0 (0)	

Abbreviation: MTHFR=methylenetetrahydrofolate reductase.

a Based on the permutation test, the *P*-value cutoff for significance was 0.009. The significant *P*-values are shown in bold cases. The *P*-values reported are uncorrected for multiple comparisons.
